# Standardizing Domains and Metrics of Stroke Recovery: A Systematic Review

**DOI:** 10.3390/brainsci14121267

**Published:** 2024-12-17

**Authors:** Yash Akkara, Ryan Afreen, Michael Lemonick, Santiago Gomez Paz, Ziad Rifi, Jenna Tosto, David Putrino, J. Mocco, Joshua Bederson, Neha Dangayach, Christopher P. Kellner

**Affiliations:** 1 School of Medicine, Imperial College London, London SW7 5NH, UK; yash.akkara22@imperial.ac.uk; 2 Icahn School of Medicine at Mount Sinai, New York, NY 10029, USA; 3 Department of Neurosurgery, Icahn School of Medicine at Mount Sinai, New York, NY 10029, USA; 4 Mount Sinai Health System, New York, NY 10029, USA

**Keywords:** stroke, recovery, rehabilitation, domain, metric, measurement, standard

## Abstract

Background and Aims: Measuring stroke recovery poses a significant challenge, given the complexity of the recovery process. We aimed to identify a standardized and data-driven set of metrics of stroke rehabilitation in the literature that ensures the inclusion of all recovery domains and subdomains in the literature. Methods: A systematic review was conducted by four reviewers using the PRISMA guidelines on PubMed, MEDLINE, and Embase for stroke recovery articles between 2004 and 2024. The inclusion criteria comprised experimental/observational studies, including ischemic and hemorrhagic stroke. All studies had ≥20 participants who were ≥18 years of age, and had a follow-up of ≥3 months. Outcomes included demographics, geographic origin, stroke mechanism, domains and subdomains, metrics used, and follow-up. A bias assessment was performed using the Newcastle-Ottawa Scale and the Cochrane Risk of Bias 2.0 tool. This study was registered with PROSPERO (CRD42024551753). Results: Our search included 324 studies with a sample of 85,156 participants. The study identified seven domains (perception, physical and motor function (PF), speech and language (S&L), cognition, activities of daily living (ADL), quality of life (QoL), and social interaction) and 96 constituent subdomains that encompass the complete landscape of the stroke recovery literature identified. The domains of PF and ADL constituted the vast share of the literature, albeit reducing in their relative representation over time, while domains such as perception and QoL have been increasingly studied since 2004. Using the domains, the study identified the set and frequency of all commonly used metrics to measure stroke recovery in the literature, of which the NIHSS (*n* = 72), BI (*n* = 55), and mRS (*n* = 51) were the most commonly used. We identified eighteen standard metrics that ensure the inclusion of all seven domains and 96 subdomains. Summary of Review and Conclusions: The identified set of domains and metrics within this study can help inform further clinical research and decision-making by providing a standardized set of metrics to be used for each domain. This approach ensures lesser represented domains and subdomains are also included during testing, providing a more complete view and measure of stroke recovery.

## 1. Introduction

Stroke is one of the leading causes of mortality and long-term disability in the United States [1,2]. Symptoms are characterized by either a cessation of blood supply to the brain or the onset of bleeding within the brain [3], leading to the two main subtypes of strokes: ischemic and hemorrhagic. About 795,000 people in the United States are affected by stroke, with 87% of the strokes characterized as ischemic and 13% as hemorrhagic [4]. Stroke significantly impacts all domains of life, leading to substantial impairments in physical and cognitive abilities, and often results in permanent loss of function. Depending on the location and extent of damage to the brain [2,3], stroke can have impacts that extend beyond the scope of its primary symptoms, affecting various aspects of an individual’s life.

There have been efforts in the literature to characterize the domains of stroke recovery. A 2009 review by Vanhook outlines three stroke domains (physical, psychological, and social), with six categories of recovery that comprise them (cognition, function, self-concept, health perception, role change, and relationships) [5]. Yet, works such as Broken Movement [6] by John Krakauer have highlighted the presence of oversimplification and a lack of longitudinal assessment in many of the standard metrics used to assess stroke recovery, with no established guides regarding how functional changes in domains ought to be classified. While there is still a lack of consensus regarding strict definitions of domains that clinicians should examine and measure in patients, the widespread effects of stroke on all aspects of life are well-known. Stroke impairs perception, directly affecting the somatosensory system by inhibiting a patient’s abilities to respond to environmental stimuli through recognition, comprehension, and differentiation [7,8]. Stroke also significantly impacts physical and motor functioning with alterations in communications between brain regions [9]. Motor impairments result in diminished day-to-day activities, contributing to socioeconomic burden and deprivation [10,11]. Reaching beyond perception and motor impairments, stroke is also a leading cause of aphasia, which impairs language processing and communication [11]. One-third of stroke patients suffer from aphasia that significantly affects speech, reading, and writing, as well as other deficits that impair functional outcomes such as emotional regulation and social participation [12,13]. Elderly stroke patients are more likely to experience limitations in their activities of daily living due to stroke-induced deficits in physical abilities, cognition, and memory [14,15]. Because of the aforementioned long-term effects and the breadth of impairments that stroke can cause, stroke also has significant effects on quality of life and interpersonal relationships [16,17].

While the etiology of stroke is multifaceted, recovery is equally complex and long-term [5]. No single stroke scale can assess all deficits or predict an accurate diagnosis for symptom management and rehabilitation [18,19]. As a result, a variety of stroke-specific metrics have been created to assess stroke severity and to help guide intervention, prevention, and management [17]. These metrics assess varying dysfunctions and recovery stages in multiple domains of disabilities. However, many of these metrics are not clinically efficient or used routinely in clinical settings [20,21]. A study by Brandler et al. shows that in 30% of stroke cases, stroke-specific metrics fail to effectively recognize stroke in clinical settings [22]. Moreover, stroke-specific metrics often do not comprehensively assess all aspects of life and health affected by stroke, providing an unsatisfactory or incomplete picture of the current health status of the patient [23,24]. While other reviews have characterized the broader domains of stroke recovery [5], their link with the metrics used to measure them and their subdomains remains understudied. While clinicians often employ commonly used stroke-specific metrics such as the National Institute of Health Stroke Scale (NIHSS) [25] or the modified Rankin Scale (mRS) [26] to help assess function and expedite care, their scope in the context of broader stroke recovery is limited [18,19]. As such, a data-driven analysis that comprehensively links domains and subdomains with the metrics that assess them would ensure clinicians can efficiently and effectively cover all aspects of stroke rehabilitation.

In all, there is a lack of comprehensive assessment regarding the usage and reporting of recovery domains and metrics in ischemic and hemorrhagic stroke in the existing literature. The objective of this study is to systematically review the literature discussing domain-specific stroke recovery to understand the landscape of evaluated domains and the metrics used for those domains to develop a standard of stroke testing that can be replicated across patients.

## 2. Methods

### 2.1. Literature Search

A structured literature search was conducted in accordance with the systematic review guidelines outlined by the Preferred Reporting Items for Systematic Review and Meta-Analysis (PRISMA) checklist [27]. Articles were searched using PubMed, MEDLINE, and Embase from January 2004 to June 2024. No restrictions or filters were used for the study design. Only articles in English were included. The keywords “Stroke Recovery OR Stroke Rehabilitation AND Domain AND Measure OR Metric OR Test OR Assessment OR Questionnaire” were used in MeSH and text-word format to retrieve journal articles. All included studies are made available in the Supplementary Material.

### 2.2. Inclusion and Exclusion Criteria

Inclusion was limited to experimental and observational cohort studies in English, consisting of patients with either ischemic or hemorrhagic stroke. Due to the differences in pathophysiology and treatment, alongside variance in the metrics used to assess recovery, patients with subarachnoid hemorrhage (SAH) were excluded from this analysis. All studies were required to have a sample size of ≥20 participants, who were all ≥18 years of age. Participants with a medical history of prior stroke, neurological impairment, or severe mental health conditions were included because of the focus on recovery domains and their assessment, which remain consistent regardless of prior medical history. No articles were excluded based on the duration following the diagnosis of stroke. All studies were required to collect recovery data longitudinally, with follow-up restricted to a minimum of 90 days following the initial/baseline measurement. In cases where a study contained a partial population that fit the inclusion criteria with sufficient details regarding their demographic details and follow-up outcomes, only subjects/treatments that were relevant to our inclusion criteria were included.

### 2.3. Data Extraction and Bias Assessment

Studies from the database search were entered into the systematic review software Covidence (Veritas Health Innovation, Melbourne, Australia). Four reviewers independently completed the initial abstract and title screening, followed by the full-text screening in accordance with the inclusion and exclusion criteria. Any conflicts in decisions were discussed until a consensus was reached with the assistance of a senior author. Data extraction was then completed based on the following set of outcomes:

Author, study name, study design, country, sample size, male:female ratio, population age (mean, median, range), mechanism of stroke, intervention (if any), metrics of stroke recovery, domains of stroke recovery, sub-domains of stroke recovery, timings of measures, follow-up, dropout rate, reason for dropout

The Newcastle-Ottawa Scale (NOS) was used for observational studies. The Cochrane Risk of Bias 2.0 tool was used for interventional studies. Quality assessment(s) were conducted independently by all reviewers (Y.A., R.A., M.L., and S.P.). Studies using the NOS were scored from 0 to 9, while studies using the Cochrane Risk of Bias 2.0 tool were scored based on a low, moderate, or high level of bias. Discrepancies in scoring were first discussed between authors and were escalated to a senior author (C.K.) in persistent conflicts, who also evaluated the study for bias to arrive at a judgment.

### 2.4. Statistical Analysis

Following the pooling of all participant demographic and treatment data, the Shapiro–Wilk test was used to establish normality, alongside the two-tailed T-test and Spearman’s R, which were used to test for the degree of correlation and regression between participant demographic factors and the domains of stroke recovery according to a fixed effects model. The Shapiro–Wilk test was used to test for normality, with the two-tailed T-test and Mann–Whitney U test being used to determine statistical significance in parametric and nonparametric datasets, respectively, within the frequency of domains, sub-domains, and metrics of stroke recovery within the literature. Any domains, sub-domains, or metrics that appeared only once in the review were excluded. The probability of each domain as a percentage of all studies in the literature was calculated by dividing the sample size of participants tested per domain across all studies, divided by the overall sample size of participants. The sample sizes of studies testing more than one domain were counted proportional to the number of individual domains tested. Data were compiled and analyzed using GraphPad Prism Version 10.1.1 for Mac (GraphPad, San Diego, CA, USA). Parametric variables are presented as mean (±standard deviation). Nonparametric datasets are presented as a median (interquartile range). *p* < 0.05 was considered statistically significant.

### 2.5. Study Registration

This review has been registered with the international prospective register of systematic reviews (PROSPERO). Registration ID: CRD42024551753.

## 3. Results

**Figure 1:**
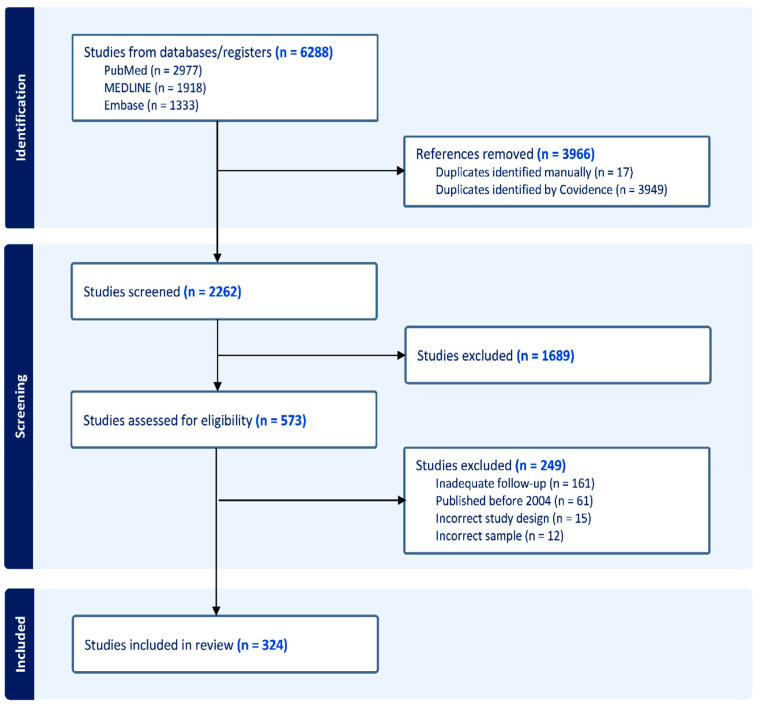
PRISMA diagram outlining the stages of screening and inclusion of studies.

A total of 6288 studies were identified within the initial search, of which 3966 articles were classed as duplicates and removed. In total, 1689 further studies were identified to be irrelevant to the study within the title and abstract screening phase. Of the 573 remaining studies that underwent full text screening, 249 studies were excluded. The reasons for exclusion included publication prior to 2004, inadequate long-term follow-up, incompatible study design, and samples that did not fit our inclusion criteria. This left 324 articles to be included within the final analysis (Figure 1).

Of the 324 articles included, 238 were observational cohort studies, 45 were longitudinal cross-sectional studies, and 41 were clinical trials, of which 31 were randomized control trials (RCT). The studies were categorized into World Health Organization (WHO) regions of origin [28], with 198 studies from the European region (EUR), 56 studies from the Western Pacific Region (WPR), 55 studies from the region of the Americas (AMR), 8 studies from the African region (AFR), and 7 studies from the South-East Asian Region (SEAR). In total, 300 of the 324 studies were from high-income countries, followed by 15 studies from upper-middle-income countries, 7 studies from lower-middle-income countries, and 2 studies from lower-income countries as classified by the World Bank. The vast majority of studies (285) included patients with both ischemic and hemorrhagic stroke, followed by 37 studies that exclusively included patients with ischemic stroke, and 2 studies solely examining patients with hemorrhagic stroke.

Across all studies, this review consisted of a sample of 85,156 patients with stroke. The mean age of all patients was 64.51 (6.63), with 49,166 (57.7%) of participants listed as male, and 36,040 (43.2%) of participants listed as female. Of the 85,156 participants, 74,265 patients (87.2%) were diagnosed with ischemic stroke, while 10,891 (12.8%) patients were diagnosed with hemorrhagic stroke. The number of patients from the regions of EUR, WPR, AMR, AFR, and SEAR was 48,633 (57.1%), 22,768 (26.6%), 12,515 (14.7%), 632 (0.8%), and 608 (0.8%), respectively.

In total, 13,241 (15.5%) patients were subjected to an explicit intervention or treatment (conservative, medical, or surgical) in addition to standard of care, while the remaining set of patients (84.5%) were observed in accordance with their prescribed management, with no explicit intervention listed in the study. Of the 13,241 patients who were subjected to a certain intervention/treatment arm across the clinical trial studies, 4263 patients received conservative management, 4326 received medical management, and 4652 received surgical management.

### 3.1. Domains

Across all 324 studies, we identified seven distinct overarching domains of stroke recovery that were present in all methods of stroke recovery testing. These seven domains were perception, physical and motor function (PF), speech and language (S&L), cognition, activities of daily living (ADLs), quality of life (QoL), and social interaction (SI), which were tested in 119 (36.7%), 253 (78.1%), 207 (63.9%), 159 (49.1%), 180 (55.6%), 83 (25.6%), and 135 (41.7%) studies, respectively.

Each domain was sub-analyzed based on the sample size of patients who were subject to such categorical testing and compared to the complete sample size, yielding the probability of a single stroke patient receiving independent testing on one of the seven domains across the literature. This acted as a surrogate indicator of how frequently each domain was represented within the literature. Across the 20-year study period, physical and motor function and ADLs were significantly more likely to be tested than any other individual domain, with probabilities of 0.32 and 0.22, respectively (*p* < 0.0001). Perception, speech and language, cognition, QoL, and social interaction had respective probabilities of 0.13, 0.12, 0.11, 0.05, and 0.05.

**Figure 2:**
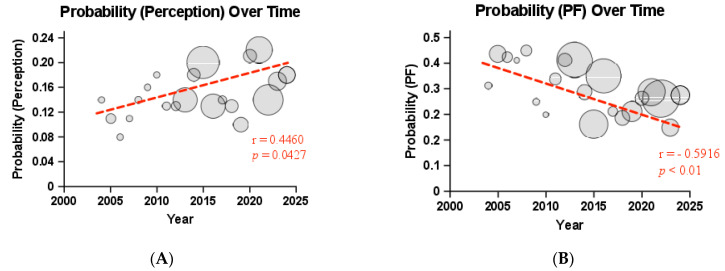

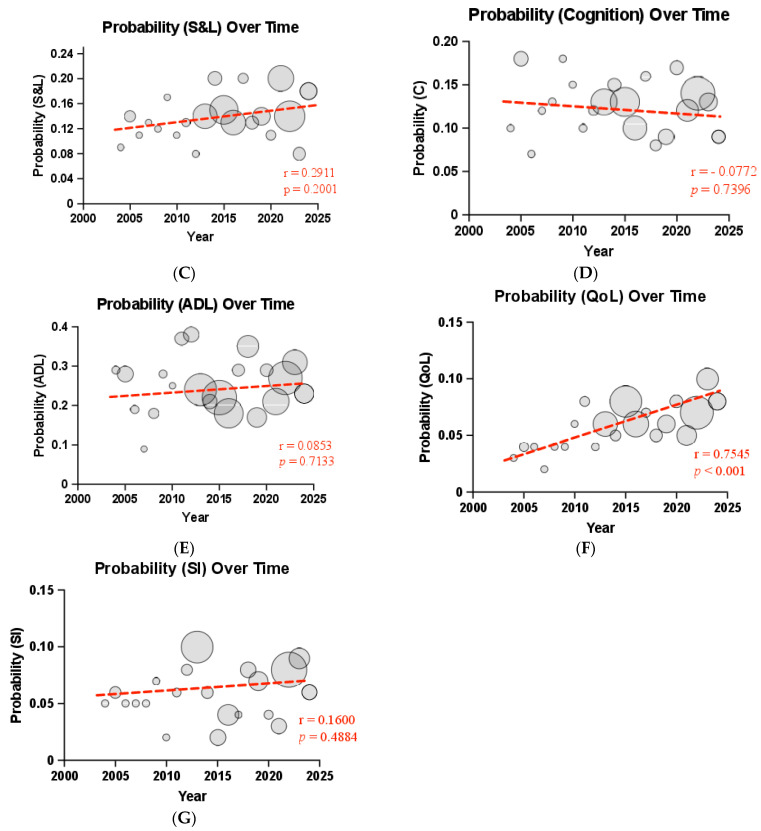
The Probability of Stroke Recovery Domains Appearing in the Literature Over Time. (**A**) Perception; (**B**) Physical Function; (**C**) Speech and Language; (**D**) Cognition; (**E**) Activities of Daily Living; (**F**) Quality of Life; (**G**) Social Interaction.

The probability of each domain’s appearance in the literature was further sub-stratified by year, yielding the probability of a patient to be tested in one of the seven domains of stroke recovery per annum. For perception, a significant increase in its probability of being measured over time was found, with the domain being represented more in the literature over time (r = 0.4460, 95% CI = [0.004079, 0.7422]). Physical and motor function experienced a significant decline in its overall representation in the literature (r = −0.5916, 95% CI = [−0.8197, −0.2017]), with other domains of stroke recovery occupying larger shares in recent years. Finally, a significant increase was also observed in the probability of QoL being measured over time (r = 0.7454, 95% CI = [0.4517, 0.8933]), with recent studies having a significantly higher representation of the domain. No significant relationship was found in the probability of speech and language (r = 0.2911, 95% CI = [−0.1741, 0.6500]), cognition (r = −0.0772, 95% CI = [−0.5027, 0.3785]), ADLs (r = 0.0853, 95% CI = [−0.3715, 0.5088]), or social interaction (r = 0.1600, 95% CI = [−0.3043, 0.5629]) being studied over time (Figure 2).

### 3.2. Subdomains

Within each of the seven larger domains, using the metrics and assessment tools used by studies, we identified a list of sub-domains that were directly or indirectly tested to gauge stroke recovery. Any subdomains that did not appear in two or more studies were excluded from the following results. This was to ensure the inclusion of subdomains that are commonly tested at a higher frequency across the literature, while more niche subdomains are excluded to prevent the need for additional standardized metrics that solely cater to less studied and more uncommon areas of recovery (Table 1).

### 3.3. Metrics

A similar analysis was performed based on the frequency of studies using various metrics used to measure stroke recovery, extending across all seven domains and underlying sub-domains. Similarly, any metric that did not appear in ≥2 independent articles was excluded from the analysis due to a lack of a sufficient evidence base for their use (Table 2).

### 3.4. Composite Measurement of Stroke Recovery

Using the domains, subdomains, and metrics corresponding to stroke recovery, we searched for the optimal set of metrics to minimize the number of assessment tools needed, while still measuring all of the domains and subdomains of stroke recovery identified in the literature. If multiple metrics met this criterion, priority was given to the more commonly studied metric. We arrived at a set of eighteen metrics of stroke recovery, which were able to measure all domains and subdomains identified throughout the literature. They are the NIHSS, BI, EQ-5D, MMSE, FMA, FIM, FAI, BBS, 6-MWT, CES-D, OCS, WMFT, GDS, LiSat, WHOQOL-BREF, ARAT, PCBS, and QAB (Table 2).

We also further divided these eighteen metrics based on the seven identified domains, allowing each domain and its respective sub-domains to be measured in their entirety using only the following metrics of stroke recovery (Table 3).

## 4. Discussion

This systematic review has identified a comprehensive set of domains, subdomains, and metrics across the literature that provide an overarching view into longitudinal stroke recovery. Through the large sample of studies included, this review aims to be representative of the entire breadth of the longitudinal stroke recovery literature.

The seven overarching domains of stroke recovery identified in this review each saw distinct rates of representation in the literature; the measurement of motor and physical function occurred in the majority of the studies, whereas QoL and social interaction emerged as the least represented domains. Several studies point to the importance of ambulation, a subdomain of motor and physical function, as a common and important focus of care following stroke, whereas other articles emphasize ADLs as the most important aspect of stroke recovery. The most common finding was an emphasis on generalized, composite “independence”, which may encompass some or all of the seven domains laid out in this review [29]. However, this finding does not provide insight into how certain metrics are employed and why, especially with regard to targeted domains or subdomains. It is presumed that the focus of stroke rehabilitation is dependent on the unique functional status of the patient, and as such, it is difficult to determine whether one domain is more important than another in the pursuit of achieving functional independence. Additionally, there is a large overlap between the domains, where drawing distinct delineations between them would prove difficult.

In addition to absolute differences in representation, the seven domains also experienced fluctuations in their relative representation in stroke literature over time. Our analysis demonstrates that while physical and motor function is still a prominent domain and nearly ubiquitously measured in stroke recovery, their share among all measures of stroke recovery has decreased since 2004. We speculate that this does not suggest that the domain of physical and motor function is being tested at a lower frequency; rather, other under-represented domains have seen an increase in their employment over the last two decades and now occupy a larger share of the literature. For example, the QoL domain experienced a significant increase in its share of the stroke recovery literature since 2004, likely reflecting an increased interest in studying other aspects of wellbeing in stroke populations. The net result is that the aggregation of stroke metrics is far more diverse than it was twenty years ago, at the beginning of our measurement period. This is due in part to the introduction of new or reformed metrics and the time it takes for them to gain widespread acceptance. Moreover, there has been a shift in the literature in recent years compared to earlier reviews toward the coverage of aspects of stroke recovery that are not strictly functional [5] (i.e., PF and ADLs), which has likely been driven by a refined understanding of the importance of emotional and social wellbeing in widespread clinical practice. This could explain the significant growth we found in domains, such as perception and QoL, alongside slight growth in the S&L and social interaction domains.

The analysis also highlights various subdomains that are highly emphasized across the literature and may, in aggregate, provide a baseline comprehensive measure of independence. The most-referenced subdomains from each of the seven overarching domains included ambulation (most commonly measured), alongside others such as bowel control, language and content, feeding, bathing, dressing, level of consciousness, and memory. The two overarching domains of ADLs and physical and motor function contained the highest concentration of highly referenced subdomains, which may indicate a preference among clinicians to prioritize recovery in these areas. It is also likely that these are the subdomains that can be most tangibly measured with a range of objective measurements, as opposed to other subdomains, such as anxiety or social participation, which may have a higher level of subjectivity [30]. Moreover, physical and functional recovery is often considered significantly more important than factors, such as QoL or social participation by clinicians, leading to a bias within the literature wherein studies measuring PF and ADLs tend to be more dominant [31]. On the other hand, we also encountered subdomains with fewer-than-expected studies representing them, such as muscle tone and spasticity (*n* = 6). Subdomains, such as spasticity, are linked with a longer symptomatic profile and recovery period than those affected more acutely by stroke [32], and hence may have been under-represented due to requirements for longer, more thorough follow-up windows. As such, while our findings comprehensively describe the landscape of stroke recovery literature and the subdomains they cover, inherent bias in the testability of domains and subdomains may affect the results.

Through the identification of the aforementioned domains and subdomains of stroke recovery and the metrics used to study them, this review was able to identify a set of eighteen metrics that, in aggregate, measure all 7 domains and 96 subdomains at least once, ensuring the inclusion of all aspects of recovery currently measured in the literature. Notably, no one metric was sufficient for the measurement of an entire domain, with each domain requiring a minimum of three separate metrics to ensure a comprehensive overview. Nevertheless, this presents a novel, data-driven understanding of how each domain of stroke recovery should be measured for patients, with clinicians being able to choose from a set of standard metrics depending on the domain of interest, reducing variability within and between patients. However, a key limitation of this approach is the lack of practicality due to the significant amount of time and effort required from both the clinician and patient to cover eighteen separate batteries/tests. An important insight here is that due to the emphasis on the inclusion of all 96 subdomains, an entire metric, and its battery may need to be administered just to cover a single, outlying subdomain. In the same vein, the metrics in each domain frequently test the same subdomains, causing an unnecessary level of repetition that, if avoided, can substantially reduce the amount of time required to comprehensively assess stroke recovery. This may be avoided by future work that focuses on each of the metrics identified in this study, with an emphasis on removing areas of repetition. This could be conducted by identifying the parts of each metric that correspond to each subdomain, following which the relevant sections from each metric can be extracted and combined with metrics from other subdomains to create a combined assessment battery with no redundancy. This would allow for an efficient yet indirectly validated test that can be directly used to assess overall domain recovery, being directly beneficial to clinicians and patients.

With regard to the metrics themselves, the NIHSS was by far the most commonly employed measurement tool for the assessment of stroke severity, especially at baseline immediately following a stroke. As the NIHSS tests a variety of critical functions following stroke, including functions in three of our seven identified domains (perception, physical and motor function, and speech and language), we expect that the NIHSS will continue to be widely employed as a key metric. It is also worth noting that the NIHSS is the only metric that spans three of our seven domains in the final set of standard metrics we identified, underscoring its broad applicability in the assessment of stroke. Other metrics that also enjoy wide applicability are BI, mRS, and the SIS, each of which is employed in more than 40 studies meeting the inclusion criteria of our evaluation. These metrics, too, cover broad aspects of stroke recovery and span various domains. Coupled with their longstanding deployment and relative ease-of-use, this allows these metrics to be utilized across a wide range of observational and interventional studies, leading to them forming significant portions of the literature. However, of these four commonly employed metrics, only NIHSS and BI feature on our list of 18 metrics to compositely measure stroke recovery, indicating that although metrics, such as mRS and SIS, are widely employed in the functional assessment of stroke, their employment may not be necessary for a comprehensive assessment of stroke as domains that fall under their purview are also measured by other metrics with larger evidence bases. On the other hand, we identified a variety of metrics that were sparingly used across the literature, many of which appeared in only a handful of studies within our overall search. Many of these metrics have been developed relatively recently, (such as the QAB), and hence have had less of an ability to disseminate across the literature, especially with their niche application in terms of subdomains. Nevertheless, some of these metrics also form a part of the 18 metrics we identified (ARAT, PCBS, and QAB), owing to their ability to identify and measure specific subdomains that older, more generalized metrics often cannot test. As such, we believe our list of standard metrics offers both a degree of ease-of-use and familiarity with its emphasis on widely used metrics, while still offering specific tests when needed for certain subdomains.

A separate review of the literature for domains of stroke recovery yielded inconclusive findings with regard to how domains are defined and described. As referenced earlier in this review, a 2009 literature synopsis by Vanhook concluded that there are three main domains of stroke recovery (physical, psychological, and social), and six categories that comprise the three domains (cognition, function, health perception, self-concept, relationships, and role change) [5]. A separate article from Braun et al. discusses how data from the NIHSS can guide clinicians and researchers in the selection of relevant domains, such as sensory, motor, or linguistic function, for further patient testing [30]. However, while this article emphasizes a domain-driven approach to outcome measures of stroke, it does not attempt to identify the most common, comprehensive stroke recovery domains, as is one of our primary conclusions from this review study. A third review, the Stroke Action Plan for Europe of the European Stroke Organisation, lists domains in terms of stroke care, as opposed to functional areas as detailed in this review [33]. Consolidating domains and subdomains of stroke recovery would provide a necessary, common framework for clinicians and deserves codification as a baseline for future stroke studies.

While this study conducted a large-scale and comprehensive search of the literature, the results may not be completely representative of global stroke patient populations. In examining demographic data derived from this review, particularly the sex and age characteristics of patients enrolled in the examined studies, we found that men were more widely included in the longitudinal studies than women. We also found that 64.51 was the average age for the study cohort. The sex distribution is not in line with the general consensus of a higher incidence of stroke in women [34,35]. The reasons for this discrepancy may include skewed sampling and inclusion that lead to a lower-than-expected proportion of women in the literature covering longitudinal stroke recovery. The average age of patients was also lower than expected, as one study of 82,774 patients notes the mean age of stroke being 74.3 [36]. The younger average age of the results from our review may be due to specific inclusion parameters of our study, such as the necessity of longitudinal tracking and data on patients, which may have led to elderly populations being disproportionately excluded as a result of a reduced likelihood of survival. In the future, we think this may be addressed by directly expanding the search criteria to include under-represented groups in the literature, ensuring the inclusion of larger samples of populations that currently form minorities in the stroke recovery literature. Future prospective work should also focus on ensuring more inclusive sampling practices, adjusting for the often indirect bias that exists against elderly populations and women, with the focus lying on recruiting participants to be as representative as possible of the distribution of the general stroke population. Moreover, focusing on the geographic distribution of studies that were included in this review, 61% of the examined studies originated from European institutions, with a subsequent focus on European patients. This was followed in number by studies from the Western Pacific Region and from the Americas. With the vast majority of studies stemming from countries, such as those in Europe, China, the United States, Canada, and Australia, alongside the paramount share of the literature arising from developed countries, the findings of this study may not be applicable to developing countries at a global scale. More research is needed in these areas to better understand publishing trends and the use of stroke metrics by nation or WHO region.

In addition to those limitations previously mentioned, the study is somewhat limited by its inclusion criteria, leading to the exclusion of a variety of stroke recovery articles, namely due to a lack of sufficient follow-up. While this was necessary for this review to ensure the usage of metrics that could be replicated and compared over a larger time scale, it led to many articles with shorter durations between measurements, (such as between admission and discharge, or discharge and one month of follow-up), to be excluded. The search criteria used may have also led to the exclusion of a large evidence base due to its focus on articles explicitly studying one or a combination of domains. It is likely that a large variety of articles were excluded that did not focus on any particular domain(s), but indirectly measured them through the use of metrics. It is also likely that there are articles with a primary focus on domains of stroke recovery, yet they were not collected in our literature search due to differences in phrasing or terminology that would evade our search terms. An additional factor that may have influenced our results is the difference between regional or national tendencies to employ specific stroke metrics instead of others. This could be driven by local policy, institutional momentum, or both. Examining regional differences in the employment of specific stroke metrics and emphasis on recovery domains provides an avenue for further research and investigation.

## 5. Conclusions

In conclusion, this analysis provides a comprehensive snapshot of the prevailing stroke metrics, their relative employment over time, the domains and subdomains that they measure, and which metrics, among the many that are employed worldwide, ultimately provide comprehensive coverage of our overarching recovery domains. Future research is merited in the investigation of each domain as well as efforts to create a new, consolidated metric to provide a comprehensive assessment to measure stroke recovery in the most efficient manner possible.

## Figures and Tables

**Table 1:** The corresponding set of subdomains to each identified domain.

Domain	Sub-Domain	Frequency (Number of Studies)
Perception	Level of Consciousness	98 (30.2%)
	Vision	82 (25.3%)
	Pain	80 (24.7%)
	Neglect	72 (22.2%)
	Orientation	55 (17.0%)
	Vitality	43 (13.3%)
	Static Balance	38 (11.7%)
	Discomfort	32 (9.9%)
	Touch	27 (8.3%)
	Proprioception	25 (7.7%)
	Dynamic Balance	15 (4.6%)
	Sleep	10 (3.1%)
	Appetite	7 (2.2%)
	Restlessness	4 (1.2%)
	Sensitivity to stimuli	4 (1.2%)
	Allodynia	4 (1.2%)
	Hearing	2 (0.6%)
Physical and Motor Function	Walking	236 (72.8%)
	Mobility	216 (66.7%)
	Bowel Control	188 (58.0%)
	Bladder Control	188 (58.0%)
	Motor–UE	167 (51.5%)
	Motor–LE	157 (48.5%)
	Motor–Face	72 (22.2%)
	Stairs	58 (17.9%)
	Dexterity	53 (16.4%)
	Joint Function	23 (7.1%)
	Fine Control	20 (6.2%)
	Coordination	18 (5.6%)
	Endurance	14 (4.3%)
	Gait	12 (3.7%)
	Lifting	12 (3.7%)
	Speed of Movements	8 (2.5%)
	Grasping	7 (2.2%)
	Tone and Spasticity	6 (1.9%)
	Gripping	2 (0.6%)
	Pinching	2 (0.6%)
	Posture	2 (0.6%)
	Comorbidities	2 (0.6%)
	Reactions	2 (0.6%)
	Apraxia	2 (0.6%)
	Drawing	2 (0.6%)
Speech and Language	Language and Content	180 (5.6%)
	Comprehension	104 (32.1%)
	Reading	82 (25.3%)
	Writing	76 (23.5%)
	Naming	61 (18.8%)
	Fluency	29 (9.0%)
	Spelling	6 (1.9%)
	Grammar	6 (1.9%)
	Repetition	4 (1.2%)
	Rate	4 (1.2%)
	Clarity	2 (0.6%)
	Responsive Speech	2 (0.6%)
Cognition	Memory	154 (47.5%)
	Attention	82 (25.3%)
	Emotion Recognition	66 (20.4%)
	Visuospatial Skills	59 (18.2%)
	Problem Solving	59 (18.2%)
	Calculation	40 (12.3%)
	Abstraction	23 (7.1%)
	Mood	21 (6.5%)
	Following Instructions	7 (2.2%)
	Planning	7 (2.2%)
	Decision Making	5 (1.5%)
	Creativity + Design	2 (0.6%)
Activities of Daily Living	Feeding	153 (47.2%)
	Bathing	153 (47.2%)
	Grooming	153 (47.2%)
	Dressing	153 (47.2%)
	Using the Toilet	153 (47.2%)
	Transfers	116 (35.8%)
	Managing Finances	58 (17.9%)
	Leisure	23 (7.1%)
	Exercise	13 (4.0%)
	Driving	13 (4.0%)
	Traveling	13 (4.0%)
	Productivity/Work	13 (4.0%)
	Sexual	6 (1.9%)
	Cooking	6 (1.9%)
Quality of Life	Depression	78 (24.1%)
	Anxiety	75 (23.1%)
	HR-QoL	53 (16.4%)
	Self-Esteem	17 (5.2%)
	Depressed Affect	6 (1.9%)
	Positive Affect	6 (1.9%)
	Coping Strategies	3 (0.9%)
	Suicidal Thoughts	3 (0.9%)
	Beliefs	2 (0.6%)
	Safety	2 (0.6%)
	Financial Security	2 (0.6%)
	Physical Environment	2 (0.6%)
	Stress	2 (0.6%)
Social Interaction	Participation	115 (35.5%)
	Interpersonal Relationships	90 (27.8%)
	Family	16 (4.9%)

**Table 2:** Metrics employed in ≥2 studies to measure stroke recovery, with their frequency of use.

Metric	Frequency
NIHSS	72 (22.2%)
Barthel Index (BI)	55 (17.0%)
Modified Rankin Scale (mRS)	51 (15.7%)
Stroke Impact Scale (SIS)	41 (12.7%)
EuroQoL 5D (EQ-5D)	29 (9.0%)
Mini-Mental State Examination (MMSE)	26 (8.0%)
Fugl-Meyer Assessment (FMA)	23 (7.1%)
Montreal Cognitive Assessment (MoCA)	23 (7.1%)
Functional independence Measure	22 (6.8%)
SF-36	19 (5.9%)
Hospital Anxiety and Depression Scale (HADS)	18 (5.6%)
Frenchay Activities Index (FAI)	13 (4.0%)
Berg Balance Scale (BBS)	10 (3.1%)
6-Minute Walk Test (6MWT)	10 (3.1%)
Activity Measure for Post Acute Care (AM-PAC)	7 (2.2%)
Neuro-QoL	7 (2.2%)
Modified Ashworth Scale (MAS)	6 (1.9%)
Center for Epidemiological Studies Depression Scale (CES-D)	6 (1.9%)
Oxford Cognitive Screen (OCS)	6 (1.9%)
Stroke Specific Quality of Life Scale (SSQoL)	6 (1.9%)
Trail Making Test (TMT)	6 (1.9%)
Utrecht Scale for Evaluation of Rehabilitation-Participation (USER-P)	6 (1.9%)
Boston Naming Test (BMT)	6 (1.9%)
Patient Health Questionnaire (PHQ-9)	6 (1.9%)
Stroke and Aphasia QoL Scale (SaQoL)	5 (1.5%)
Wolf Motor Function Test (WMFT)	5 (1.5%)
Functional Ambulatory Category (FAC)	5 (1.5%)
Geriatric Depression Scale (GDS)	4 (1.2%)
Life Satisfaction Questionnaire (LiSat)	4 (1.2%)
Visual Analogue Scale and Leeds Assessment of Neuropathic Symptoms and Signs pain scale (VAS-LANSS)	4 (1.2%)
Instrumental Activities of Daily Living (IADL)	4 (1.2%)
Patient-Reported Outcomes Measurement Information System (PROMIS)	4 (1.2%)
Brixton Spatial Anticipation Test (BSAT)	4 (1.2%)
Activity Card Sort (ACS)	3 (0.9%)
Montgomery Asberg Depression Scale (MADRS)	3 (0.9%)
Short Physical Performance Battery (SPPB)	3 (0.9%)
WHODAS 2.0	3 (0.9%)
WHOQOL-BREF	3 (0.9%)
Fatigue Severity Scale (FSS)	3 (0.9%)
Occupational Gaps Questionnaire (OGQ)	3 (0.9%)
Reintegration to Normal Living Index (RNLI)	3 (0.9%)
General Health Questionnaire 12 (GHQ-12)	3 (0.9%)
Lawton and Brody’s 8-point scale	3 (0.9%)
Wechsler Adult Intelligence Scale (WAIS)	3 (0.9%)
Wechsler Memory Scale (WMS)	3 (0.9%)
Action Research Arm Test (ARAT)	2 (0.6%)
Assessment of QoL Scale (AQoL)	2 (0.6%)
Charleston Comorbidity Index (CCI)	2 (0.6%)
Postural Control and Balance for Stroke (PCBS)	2 (0.6%)
Quick Aphasia Battery (QAB)	2 (0.6%)
Repeatable Battery of Neuropsychological Status (RBANS)	2 (0.6%)
Western Aphasia Battery (WAB)	2 (0.6%)
Motricity Index	2 (0.6%)
Activity Specific Balance Confidence Scale (ABC)	2 (0.6%)
Apathy Evaluation Scale	2 (0.6%)
Nottingham Extended Activities of Daily Living (NEADL)	2 (0.6%)
Pain Catastrophizing Scale (PCS)	2 (0.6%)
Profile of Mood States	2 (0.6%)
Informant Questionnaire on Cognitive Decline in the Elderly (IQCODE)	2 (0.6%)
Corsi Block Tapping Test	2 (0.6%)

**Table 3:** The set of identified metric standards corresponding to each domain of recovery.

Domain	Metrics (Bold = Used in More Than One Domain)
Perception	**NIHSS, EQ-5D, MMSE, FMA**, BBS, **GDS, WHOQOL-BREF, ARAT**
Physical and Motor Function	**NIHSS, BI, FMA**, 6MWT, WMFT, PCBS, **ARAT**
Speech and Language	**NIHSS, MMSE**, QAB
Cognition	**MMSE, FIM**, OCS
ADLs	**BI,** FAI, **LiSat**
QoL	**EQ-5D, CES-D, GDS, LiSat, WHOQOL-BREF**
Social Interaction	**FIM, CES-D, LiSat**

## Data Availability

All included studies are provided within the Supplementary Material. Further enquiries can be directed to the corresponding author.

## Supplementary Materials

The following supporting information can be downloaded at: https://www.mdpi.com/article/10.3390/brainsci14121267/s1, S1 (The list and authors of all 324 included studies).
